# Hyoscine-n-butylbromide in treating abdominal pain caused by gastroenteritis: a double-blind randomized placebo-controlled study

**DOI:** 10.55730/1300-0144.5864

**Published:** 2024-06-25

**Authors:** Çağdaş YILDIRIM, Gül PAMUKÇU GÜNAYDIN

**Affiliations:** 1 Department of Emergency Medicine, Faculty of Medicine, Ankara Yıldırım Beyazıt University, Ankara, Turkiye; 2 Department of Emergency Medicine, Ankara Bilkent City Hospital, Ankara, Turkiye

**Keywords:** Butylscopolammonium bromide, abdominal pain, gastroenteritis, pain management

## Abstract

**Background/aim:**

Hyoscine-N-butylbromide (HBB) is an anticholinergic agent widely used to treat pain caused by spasms in the gastrointestinal and urogenital systems. The aim of this study was to compare the efficacy of HBB with a placebo in treating abdominal cramping pain caused by acute gastroenteritis in the emergency department (ED).

**Materials and methods:**

This was a prospective, double-blind, placebo-controlled, randomized trial conducted in a single-center academic ED from September to November 2021. Patients aged 18–65 years with acute gastroenteritis symptoms were included. The study compared the efficacy of intravenous HBB (20 mg) to a placebo. The primary outcome was the absolute change in pain score at 30 min after treatment, with secondary outcomes including pain relief at 60 min, adverse events, and the need for rescue analgesics.

**Results:**

Fifty patients were randomized (25 in each group). There was no significant difference in 30-min and 60-min pain scores between the groups. At 60 min, pain reduction and the need for rescue analgesia were similar in both groups. Changes in pain scores from admission to 30 and 60 min did not significantly differ between the groups.

**Conclusion:**

Intravenous HBB did not show a statistically or clinically significant difference in pain reduction compared to a placebo in patients with acute gastroenteritis and cramping abdominal pain in the ED.

## 1. Introduction

Hyoscine-N-butylbromide (HBB) is an anticholinergic hyoscine derivative obtained from the leaves of *Duboisia* trees found in South America and Australia. HBB, also known as scopolamine-N-butylbromide, N-butylscopolammonium bromide, and butyl scopolamine, blocks acetylcholine activity at both muscarinic and nicotinic receptors and acts on gastrointestinal (GI) smooth muscle, cardiac muscle, and exocrine gland cells, including GI epithelial cells [1,2]. Hyoscine-N-butylbromide is used to reduce motility in the gastrointestinal and urogenital systems and to treat pain caused by spasms in these systems due to its smooth-muscle relaxant and spasmolytic effects. Side effects of HBB (blurred vision, palpitations, constipation, dry mouth, hypotension, dizziness, urinary retention) are frequent but usually minor and self-limiting [1].

HBB is commonly used to treat nonspecific colicky abdominal pain. It decreases GI tract motility by binding to muscarinic receptors in visceral smooth muscle and shows parasympathetic ganglion blocking effects by binding to nicotinic receptors [2]. In emergency medicine practice, HBB is used to treat conditions such as gastroenteritis, colitis, irritable bowel syndrome, renal colic, biliary colic, primary dysmenorrhea, and nonspecific abdominal pain [1].

Gastroenteritis is an inflammation of the stomach, small intestine, or large intestine. Along with upper respiratory tract infection, it is the most common infectious disease in the world [3,4]. Acute gastroenteritis (AGE) usually lasts less than 14 days [4]. Symptoms include abdominal cramping, pain, nausea, vomiting, and diarrhea. The typical initial symptom of the leading causes of gastroenteritis, such as traveler’s diarrhea, *Clostridium difficile* infection, and giardiasis, is abdominal cramping pain [3,5]. There are studies in the literature on the use of HBB in the management of pain of smooth-muscle origin (e.g., renal colic, biliary colic, or inflammatory bowel disease) [1,2,6]. Although HBB is used in daily practice in emergency medicine for nonspecific abdominal pain and abdominal cramping pain caused by gastroenteritis, we did not find any previous studies comparing the efficacy of HBB with a placebo in abdominal cramping pain caused by AGE in our literature review.

The aim of this study was to compare the efficacy of HBB with a placebo in the treatment of abdominal cramping pain caused by AGE in an emergency department (ED).

## 2. Materials and methods

This single-center, prospective, double-blind, placebo-controlled, randomized trial was carried out with patients who had AGE. Results were reported according to the Consolidated Standards of Reporting Trials guidelines. The study was conducted from September to November 2021 in an academic ED with approximately 240,000 patient visits per year. The efficacy and safety of intravenous (IV) HBB were compared with normal IV saline in the acute treatment of abdominal cramping pain in AGE. This study was performed in accordance with the tenets of the Declaration of Helsinki and approval was obtained from the local ethics committee. The study was registered at clinicaltrials.gov (NCT04682860/14.12.2020). Written informed consent was obtained from all patients before their enrollment in the study.

Patients between 18 and 65 years who presented to the ED with watery stools that had started in the last 2 weeks were evaluated. Patients who had passed at least 3 stools in the last 24 h and had abdominal pain were included in the study [5,7]. Patients were excluded if they refused to give informed consent; were unable to understand or mark scores on the visual analog scale (VAS); had received any analgesic drugs within 4 h before the ED visit; had any hemodynamic abnormalities (systolic blood pressure of <100 mmHg, heart rate of >100/ min), peritonitis/acute abdomen signs on physical exam, documented allergy to the study drugs, diabetes mellitus, or other neuropathic diseases that alter pain perception; were given any medications in the ED before being included in the study; were pregnant; or had a final diagnosis in the ED unlikely to involve AGE. Patients were also excluded if they had a condition for which HBB parenteral administration was contraindicated (i.e., patients with untreated narrow angle glaucoma, tachycardia, hypertrophy of the prostate with urinary retention, mechanical stenoses of the gastrointestinal tract, myasthenia gravis, and Hirschsprung’s disease).

The randomization schedule was generated with a computer-based program (http://www.randomization.com). Eligible patients were randomly assigned in a 1:1 ratio to receive either a single IV dose of 1 mL of HBB (20 mg) mixed with 1 mL of normal saline or 2 mL of normal saline. To ensure the blinding of the study and to ensure equal numbers of patients in the study arms, blocks of 10 were used in the randomization scheme. The placebo and treatment injections were prepared beforehand by a nurse who was not involved in any part of the care of the participants. Both treatment and placebo IV solutions were administered in 30 s. The patients were blinded to the medication they received. If a patient’s pain was not adequately controlled by the first medication (placebo or HBB) at 30 or 60 min, rescue analgesia was administered. Dexketoprofen administration (IV, 50 mg) was used for rescue analgesia.

The enrollment period continued for 24 h a day and senior residents received training about the study protocol and the relevant World Health Organization (WHO) criteria before the start of the study. All ED patients were assessed for AGE at presentation according to the WHO criteria [5]. After selection of an eligible patient by a senior emergency medicine resident, the patient was asked to sign an informed consent form to participate in the study and was assigned a number from the randomization scheme by the senior resident. The nurse gave the already prepared and previously numbered IV HBB solution or IV saline solution to the treating physician, who administered it to the patient. The nurses and physicians who were involved in the selection and treatment of the patients and the statistician were blinded to the treatment.

Each patient allocated to one of the study groups was first asked by the physician to describe the intensity of the abdominal pain using a 10-cm VAS score. The IV study drug was then administered by the physician and two additional VAS scores were recorded at 30 and 60 min. The timing of rescue analgesia (if needed) was recorded on the data collection sheet.

The primary outcome measure was the absolute change in pain score at 0 and 30 min between the groups. The secondary outcome measures were pain relief at 60 min, any adverse events, and the need for rescue analgesics in the ED.

All statistical analyses were performed using IBM SPSS Statistics 20.0 for Windows (IBM Corp., Armonk, NY, USA) and Stata/SE version 12.0 (Stata Corp., College Station, TX, USA). Intention-to-treat analysis was performed. Demographic data and frequency distributions were first analyzed. Pearson chi-square tests and Fisher exact tests were used for ratio comparisons of ordinal data between groups. Normality analysis of continuous (numerical) data was performed using the Shapiro–Wilk test. When two independent groups were compared, the Mann–Whitney U test was used for parameters that did not show normal distribution and the independent samples t-test was used for parameters that showed normal distribution. Values of p < 0.05 were considered statistically significant. All statistical analyses were two-sided. The sample size was estimated with G*Power for Mac OS X (version 3.1.9.2; University of Düsseldorf, Düsseldorf, Germany). Our goal was to achieve power to detect a 13-mm difference on the VAS according to the study by Todd and Funk [8]. In sample size analysis based on a baseline VAS score of 5.1 cm in the study by Mueller-Lissner et al. and assuming a VAS difference of 1.3 cm as clinically significant, it was calculated that at least 18 patients should be included in each group with 80% power and 5% type-1 error [9]. According to this calculation and considering possible missing data, 25 patients were planned to be included in each group.

## 3. Results

A total of 76 patients were assessed for eligibility according to the WHO criteria for diarrhea and 16 patients were excluded from the study. Ultimately, 50 patients were randomized into two groups (25 patients for each study group). Of these, one patient in the treatment group and two in the placebo group discontinued participation after the measurement at 30 min. These three patients were included in the statistical analysis (Figure). Demographic features of the patients are shown in Table 1.

There was no statistically significant difference between the mean VAS scores of the groups at baseline. There was no statistically significant difference between the means of VAS scores at 30 and 60 min (Table 2). The mean 30-min VAS score was 35.3 ± 28.8 in the treatment group and 36.2 ± 23.0 in the placebo group; this difference was not statistically significant (p = 0.871; Table 2).

At 30 min, the side effect of dizziness was observed in two (8.0%) patients in the treatment group and one (4.0%) patient in the placebo group; four (16.0%) patients in each group needed rescue analgesic medication (p = 1.000).

In 47 (94.0%) patients, the median VAS score at 60 min was 10.0 (2.0–28.0) in the treatment group compared to 16.0 (1.0–42.0) in the control group; however, the difference was not statistically or clinically significant (p = 0.310; Table 2). No side effects were detected in any patient at 60 min. At 60 min, a total of five patients, three (13.0%) in the placebo group and two (8.3%) in the treatment group, required rescue analgesic medication (p = 0.666).

Changes between VAS scores at admission and at 30 and 60 min were compared. There were no statistically significant differences between the changes in VAS scores measured at admission or 30 min between the groups (p = 0.197). Considering the change in VAS values measured at 0 and 60 min, the mean difference was 39.1 ± 24.5 in the treatment group and 29.5 ± 26.1 in the placebo group. There was a difference of 9.6 mm between the mean changes between 0 and 60 min for the VAS scores of the two groups, but this difference was not statistically significant. No statistically significant difference was found between the groups in terms of VAS score differences in these three time periods (Table 3).

## 4. Discussion

HBB is an antispasmodic that has been on the market since 1952. The most well-known brand name in the world is Buscopan and it is a frequently used agent in ED treatments due to its antispasmodic effects. The fact that it is generally safe and inexpensive has popularized its use [1,10]. The absorption of HBB after oral administration is about 8% and systemic bioavailability is 1%. Therefore, systemic anticholinergic effects are not expected when it is given orally [1,2]. It is rapidly distributed into tissues when given in IV form (t_1/2_ = 29 min). When given parenterally, anticholinergic effects related to the central nervous system are not observed since it does not pass into the central nervous system [1]. In our study, HBB was administered in IV form, and in accordance with the literature, the frequency of side effects was low and there was no difference between the study and placebo groups in terms of side effects. The use of HBB in cases of nonspecific abdominal colic or abdominal pain is based on the smooth-muscle relaxation and resolution of spasms/cramps due to its antimuscarinic effects on the smooth muscles of the gastrointestinal tract [1,2,6]. Studies investigating the effect of HBB on abdominal cramping or abdominal pain have generally been conducted for diseases involving chronic abdominal cramping such as IBD or for nonspecific undiagnosed abdominal pain and with the oral or rectal form of the drug. In those studies, the effects of the drug on symptom intensity over a long period of time were measured, not the acute effects of the drug [2,11]. In most of those studies, oral HBB alone or in combination with paracetamol was shown to reduce cramping abdominal pain. For example, in a study of 1637 patients with nonspecific recurrent colic/cramping abdominal pain, HBB at 10 mg orally three times a day, paracetamol at 3 × 500 mg orally, and a combination of both were compared with a placebo. Pain was reduced at levels clinically and statistically significantly higher in all three groups compared to the placebo [9].

Muscarinic acetylcholine receptors are involved in various functions related to intestinal epithelial homeostasis in addition to previously known intestinal contractions and secretions [12]. Another less appreciated effect of HBB, associated with its antimuscarinic effect, is its antisecretory property. Therefore, we thought that HBB might be useful for treating secretory diarrhea. Based on in vitro studies, it was proposed that HBB has a local effect at the intestinal wall level when taken orally. Those results were supported by in vitro studies comparing the effect of the drug administered at the mucosa side to the effect of the drug administered at the serosa side. Antimuscarinic effects were observed in both cases, although the concentration required to achieve those effects was higher when HBB was administered at the serosa side [11]. This may be the reason why we did not observe any statistically significant changes between IV placebo and IV HBB. The effect of higher IV doses of HBB should be studied.

After IV administration of 20 mg HBB, a maximal pharmacological effect is reached at 2–15 min and the effects are expected to wane off completely after approximately 40 min [6]. According to a review, in most clinical studies the recommended dose of IV HBB is 20–40 mg. Single-dose HBB onset of action is 10 min, and it is effective at 30 min in relieving renal colic and biliary colic pain [6]. In a study by Américo et al., 20 mg of IV HBB decreased the mechanical motility index in the stomach by 50% [13]. For this reason, we chose IV HBB at a dose of 20 mg in our study, but we did not find any difference in pain reduction between the HBB and placebo groups.

Studies showing the effects of IV HBB on cramping abdominal pain due to AGE when given in the ED are insufficient. We found only one study that investigated the effect of HBB on abdominal pain as a result of AGE. In that study, HBB and paracetamol were used for the symptomatic treatment of abdominal pain. A clinically significant reduction in pain and cramps was detected in both patient groups. They found that both drugs had a similar effect on reducing pain. There was no difference between the two drugs in terms of treatment efficacy or side effects. No severe side effects were observed in any of the patients in either group, but drug effect was not measured against the placebo [14].

Remington et al. compared HBB and paracetamol in the management of patients with undifferentiated abdominal pain presenting to the ED. The results of that trial suggested that paracetamol alone may be used in the treatment of patients presenting to the ED with mild to moderate acute undifferentiated abdominal pain. That study involved three groups (HBB + placebo, paracetamol + placebo, and HBB + paracetamol); thus, there was no placebo-only arm in that study [15].

Our study differed from the two aforementioned studies in comparing the effect of IV HBB solely with a placebo in abdominal pain due to AGE. As in the previous studies, a statistically significant decrease in pain was observed in both groups in our study, but we found no difference between the HBB and placebo in terms of reduction in pain or the need for rescue analgesia.

In this randomized controlled clinical trial, we found that HBB was not superior to a placebo for pain control in patients with AGE and cramping abdominal pain in the first 60 min after administration in the ED.

Among the limitations of this study, the patients were not followed after 60 min. Thus, this study does not provide information about the duration of any abdominal pain that resumed after 60 min or whether there was a need for retreatment. Another limitation is the fact that the study was a single-center study. The population over 65 years of age was excluded because of difficulties in the differential diagnosis of abdominal pain.

In this randomized controlled clinical trial, we found that in patients with AGE and cramping abdominal pain, HBB reduced pain within the first 60 min after administration in the ED, but this effect was not different from that of a placebo. There was no statistically or clinically significant difference in pain reduction between the placebo and HBB groups.

## Figures and Tables

**Figure:**
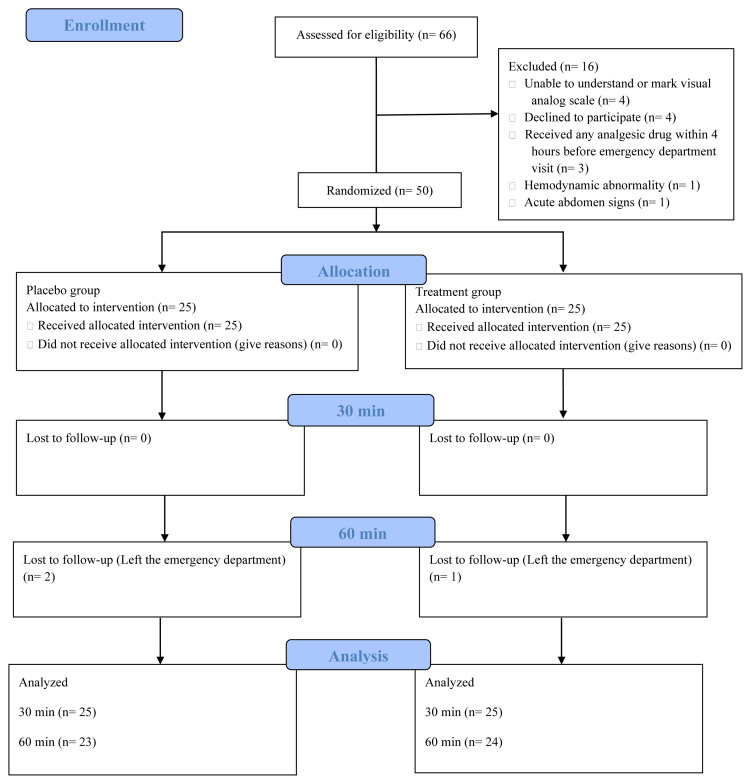
Flowchart of the study.

**Table 1:** Demographics of the study groups.

Parameters		Main study groups						
		Placebo group (n = 25)			Treatment group (n = 25)			p-value
		n (%)	Mean ± SD (95% CI)	Med. (25/–75%)	n (%)	Mean ± SD (95% CI)	Med. (25–75%)	
Sex	Female	13 (52.0)			16 (64.0)			0.390^a^
	Male	12 (48.0)			9 (36.0)			
Age (years)			33.6 ± 8.8 (29.7–37.4)	34.0 (28.0–39.0)		28.6 ± 8.8 (24.9–32.4)	26.0 (23.0–31.0)	0.008^b^

a Pearson’s chi-squared test,

b Fisher’s exact test,

SD: standard deviation, CI: confidence interval, Med.: median.

**Table 2:** Outcomes of the study groups.

Parameters		Main study groups						
		Placebo group (n = 25)			Treatment group (n = 25)			p-value
		n (%)	Mean ± SD (95% CI)	Med. (25%–75%)	n (%)	Mean ± SD (95% CI)	Med. (25%–75%)	
VAS-0 (mm)			53.2 ± 22.1 (43.7–62.8)	59.0 (33.0–66.0)		57.0 ± 19.0 (49.0–65.1)	60.0 (43.0–68.0)	0.440^a^
30th min - adverse rxn	No	24 (96.0)			23 (92.0)			1.000^b^
	Dizziness	1 (4.0)			2 (8.0)			
30th min - rescue drug	No	21 (84.0)			21 (84.0)			1.000^b^
	Yes	4 (16.0)			4 (16.0)			
30th min - proceeding	No	0 (0.0)			0 (0.0)			-
	Yes	25 (100.0)			25 (100.0)			
VAS-30 (mm)			36.2 ± 23.0 (26.2–46.1)	37.0 (13.0–53.0)		35.3 ± 28.8 (23.1–47.4)	30.0 (12.0–54.0)	0.871^b^
60th min- adverse rxn	No	23 (100.0)			24 (100.0)			-
	Yes	0 (0.0)			0 (0.0)			
60th min- rescue drug	No	20 (87.0)			22 (91.7)			0.666^c^
	Yes	3 (13.0)			2 (8.3)			
60th min- proceeding	No	2 (8.0)			1 (4.0)			1.000^c^
	Yes	23 (92.0)			24 (96.0)			
VAS-60 (mm)			23.8 ± 23.1 (13.8–33.8)	16.0 (1.0–42.0)		17.9 ± 22.7 (8.3–27.5)	10.0 (2.0–28.0)	0.310^a^

a Mann–Whitney-U test,

b Independent samples t-test,

c Fisher’s exact test,

SD: standard deviation, CI: confidence interval, Med.: median, VAS: visual analog scale, mm: millimeter, min: minute, rxn: reaction.

**Table 3:** VAS differences between time periods.

Parameters	Main study groups		
	Placebo group	Treatment group	p-value a
	Mean ± SD (95%CI)	Mean ± SD (95%CI)	
VAS diff. 0–30 (mm)	17.0 ± 20.7 (8.1–26.0)	21.8 ± 22.3 (12.4–31.2)	0.604
VAS diff. 0–60 (mm)	29.5 ± 26.1 (18.2–40.8)	39.1 ± 24.5 (28.8–49.5)	0.197
VAS diff. 30–60 (mm)	12.4 ± 14.5 (6.2–18.7)	17.3 ± 24.2 (7.1–27.6)	0.408

a Independent samples t-test,

SD: standard deviation, CI: confidence interval, VAS: visual analog scale, mm: millimeter, diff.: difference.
